# Molecular Markers for the Phylogenetic Reconstruction of *Trypanosoma cruzi*: A Quantitative Review

**DOI:** 10.3390/pathogens14010072

**Published:** 2025-01-14

**Authors:** David Ramírez-Delgado, Carlos Alberto Flores-López

**Affiliations:** 1 Independent Researcher, Ensenada 22860, BC, Mexico; linfocitoth1@gmail.com; 2 Facultad de Ciencias, Universidad Autónoma de Baja California, Ensenada 22860, BC, Mexico

**Keywords:** *Trypanosoma cruzi* phylogenetics, discrete typing units (DTUs), phylogenetic resolution, *Trypanosoma cruzi* molecular markers, *Trypanosoma cruzi* genetic diversity, phylogenetically informative characters

## Abstract

*Trypanosoma cruzi* is the parasite responsible for Chagas disease, which has a significant amount of genetic diversification among the species complex. Many efforts are routinely made to characterize the genetic lineages of *T. cruzi* circulating in a particular geographic area. However, the genetic loci used to typify the genetic lineages of *T. cruzi* have not been consistent between studies. We report a quantitative analysis of the phylogenetic power that is acquired from the commonly used genetic loci that are employed for the typification of *T. cruzi* into its current taxonomic nomenclature. Based on three quantitative criteria (the number of phylogenetic informative characters, number of available reference sequences in public repositories, and accessibility to DNA sequences for their use as outgroup sequences), we examine and discuss the most appropriate genetic loci for the genetic typification of *T. cruzi*. Although the mini-exon gene is by far the locus that has been most widely used, it is not the most appropriate marker for the typification of *T. cruzi* based on the construction of a resolved phylogenetic tree. Overall, the mitochondrial COII-NDI locus stands out as the best molecular marker for this purpose, followed by the Cytochrome b and the Lathosterol oxidase genes.

## 1. Biological Rationale for Genetically Characterizing *Trypanosoma cruzi*

*Trypanosoma cruzi* is a kinetoplastid parasite that requires two species to complete its life cycle [1]. It has been described in at least nine orders of mammals in the American continent [2]. *T. cruzi* is a heteroxenous parasite, requiring two different hosts to complete its life cycle. It requires a vertebrate host (primarily mammals of the orders Marsupialia, Rodentia, and Carnivora) and a vector invertebrate host (species within the Triatominae subfamily). The hematophagous triatomines become infected when they feed on an infected vertebrate host. Inside the triatomines, the parasite multiplies within the hindgut. Subsequently, the parasite is transmitted to a vertebrate host by parasites present in the feces of the vector if they enter either via contact with the bite site or through a mucosa (e.g., through the conjunctiva or mucous). Alternatively, a mammal can become infected if the mammal ingests the infected vector [3]. Inside the mammal host, the parasite develops intracellularly, where it has the potential to invade a wide a variety of tissues, eventually rupturing these cells and releasing parasites into the bloodstream. In the bloodstream, the parasite can infect new tissues within the mammal or be picked up by a triatomine vector feeding on that host to continue its life cycle [4].

Molecular clock analyses have estimated that *T. cruzi* basal genetic lineages started diverging approximately two million years ago [5], which is in accordance with the significant amount of genetic divergence observed between strains [6,7,8,9,10,11,12,13].

This large genetic diversity observed within *T. cruzi* is potentially a result of the parasite’s broad distribution of hosts and habitats, leading to particular genomic adaptations. Although a clear picture of the actual evolutionary history of how this adaptation took place has not been revealed, there is a likelihood that if different evolutionary lineages of the parasite evolved within different niches (i.e., hosts and vectors), this would have led to specific genomic adaptations unique to each lineage. Alongside the neutral genetic divergence expected of predominantly clonal populations, these factors most likely contributed to the significant current genetic divergence observed within *T. cruzi* lineages.

Concerning the genetic differences that have been identified in *T. cruzi*, these appear to have certain biogeographical patterns [8,14,15,16,17,18,19]. Due to these patterns, it has been hypothesized that there could be a correlation between the genetic background of the parasite and the diverse pathological outcomes of Chagas disease. However, this association remains to be determined since only a handful of studies have found a potential correlation [19,20,21,22,23], while others have not [14,17]. Larger studies are needed to evaluate this. Nonetheless, the different pathological outcomes that can be developed from an infected patient have been well studied.

Within a human host, the parasite can infect multiple tissues and cause various pathologies. The disease classically presents itself with a short incubation period (1 to 2 weeks). After this, the acute phase begins, lasting 8 to 12 weeks. Although parasitemia is present, in most cases, it remains asymptomatic or mild, and non-specific symptoms are observed (fever, malaise, anorexia, headache, diarrhea, myalgia, and lymphadenopathy) [24,25,26]. The Romaña’s sign could also be presented if the conjunctiva was the entry point [24]. In total, the severe acute phase occurs in less than 5% of cases and resolves spontaneously [25]. Nevertheless, 30% of untreated patients progress to a chronic phase, which can be categorized into indeterminate, cardiac, digestive, or neurological forms, the latter occurring in sporadic cases [27,28]. Patients in the indeterminate form remain asymptomatic for a prolonged period (e.g., decades) [24]. Regarding the genetic diversity of *T. cruzi*, this pathological outcome of Chagas disease is the most common form observed in Brazil. However, the cardiac and cardio-digestive forms are less frequent [15]. On the other hand, in the chronic forms, patients exhibit cardiovascular and digestive symptoms driven by an exacerbated inflammatory immune response. The digestive form involves the megaesophagus or megacolon observed in severe cases. As mentioned above, these presentations of Chagas disease are observed in Argentina, Brazil, Bolivia, Chile, Paraguay, and Uruguay but are virtually absent in Venezuela and Central America [15,19,27,29]. However, chronic cardiac manifestations are commonly observed in patients born in Argentina, Bolivia, Colombia, Venezuela, and Brazil [21].

As previously mentioned, there is no consensus on a possible correlation between the genetic background of *T. cruzi* and the diverse array of pathologies linked with Chagas disease. Furthermore, recent approaches to explain the complexity of the outcomes of Chagas disease involve the host–vector–pathogen tripartite interaction complex [13,15,27]. However, the genetic description of a biogeographical correlation has been thoroughly described [13,18,19,23,30], which gives grounds for the typification of the genetic lineages of this important human parasite.

## 2. Taxonomic Classification of *Trypanosoma cruzi*

*Trypanosoma cruzi* was described more than one hundred years ago by Carlos Chagas [1], and advances in molecular technologies have enabled its classification with greater precision. Since morphological differences are not apparent between different *T. cruzi* lineages, a molecular description is needed to identify the different genetic lineages of the parasite. The first attempt to classify *T. cruzi* with a molecular approach was based on the enzyme profile obtained by comparing the electrophoresis pattern retrieved from multiple enzymes of particular *T. cruzi* strains, given the fact that different strains have different alleles for the same enzymes and hence produce different protein bands when separated in electrophoresis by size and charge. This technique, called isoenzyme analysis, resulted in *T. cruzi* being classified into zymodemes (Z1, Z2 and Z3) [31]. Morel et al. [32] was the first to use the term schizodemes to classify *T. cruzi*. This classification was based on the restriction fragments that are obtained from the amplification of the *T. cruzi* minicircle gene. Following the schizodeme characterization, a more sensitive way to study kDNA polymorphism was the low-stringency single specific primer–PCR (LSSP-PCR) method, developed by Vago et al. [33]. In parallel, additional techniques were employed, such as the random amplified polymorphic DNA (RAPD) technique [10,11,34,35,36]. This approach was consistent with the description of two major lineages previously identified through the zymodeme analysis [31,37]. Moreover, with the development of molecular biology technologies, different approaches were applied to solve the elusive classification of this infectious agent. Eventually, the polymorphism of a specific messenger RNA (mRNA) sequence, known as mini-exon, was employed. The mini-exon gene is a trypanosomatid unique mRNA transcript [7,11,15,38]. Initially, the use of the mini-exon allowed the clustering of *T. cruzi* strains into two groups: Group 1, which yielded 300 bp PCR amplicons, and Group 2, which yielded 350 bp amplicons [11]. The subsequent analysis of Z3 strains identified a sub-clade inside group 2 [7]. Notably, trypanosomatids possess a unique organization of their ribosomal RNA (rRNA). Both the small and large subunits of their rRNA are significantly larger than those found in typical eukaryotic species. Within this organization, the small and large subunits are separated by two internal transcribed spacers (ITS1 and ITS2), which have been used to differentiate *T. cruzi* strains, as well as the products of 24SDNA rRNA [11,15]. The effort to demonstrate the phylogenetic relationships between the various strains of *T. cruzi* has employed other strategies, such as karyotypic comparisons [39,40]. However, the discovery of microsatellites provided another method for analyzing the *T. cruzi* population structure and phylogenetic relationship strains [9,41].

Overall, even though a diverse array of techniques have been employed to classify *T. cruzi* strains (MLEE, rRNA, mini-exon, and karyotype), the outcome has not been consistent. Subsequently, once DNA sequencing technologies were developed, the window was opened for researchers to start analyzing DNA sequence data. At the molecular level, DNA sequence analysis always provides the highest resolution and, in most cases, offers insights into resolving classification issues. So, it is not surprising that the DNA sequence of *T. cruzi* was also sought, and as a result, the first DNA sequences for *T. cruzi* were analyzed, and thus, data with a higher resolution to unravel the genetic background of *T. cruzi* strains were adopted. These first studies focused on reconstructing the evolutionary history of *T. cruzi* based on constructing a phylogenetic tree from ribosomal RNA sequences and the mini-exon gene [11,42]. This effort initially classified *T. cruzi* into two major groups designated as *T. cruzi* I and *T. cruzi* II [6,11,43]. Subsequent analyses subdivided TcII into five subgroups (IIa, IIb, IIc, IId, and IIe) [11,44]. However, more refined phylogenetic analysis revealed TcII to be a paraphyletic group [45], and thus, the division of *T. cruzi* into two major lineages was eventually modified. As a result of these efforts to classify *T. cruzi*, a novel nomenclature was introduced [17]. The updated classification scheme was based on a multilocus genotyping approach, which divided the genetic diversity into six groups named “Discrete Typing Units” (DTUs), which include TcI, TcII, TcIII, TcIV, TcV, and TcVI [46]. Furthermore, based on thorough phylogenetic analyses, the current classification status based on six DTUs has been challenged, where an alternative classification based on three mitochondrial clusters appears to be more consistent with DNA sequence data [47]. Nonetheless, since the six-DTU classification system was established, additional lineages have been discovered (TcBat and TcIV-USA), suggesting the true genetic diversity of the parasite will become greater once more exhaustive sampling is conducted [48,49]. Consequently, a new upgraded classification system should not be discarded.

In view of the current classification system, the methodology applied to identify the DTU of a particular *T. cruzi* sample usually requires the PCR amplification of a genetic locus (locus), followed by the comparison of the pattern obtained from the particular locus with reference sequences. Some methods exclude the need to determine the DNA sequence of the particular locus used for the typification of the parasite [50,51]. For example, the commonly used mini-exon locus consists of typifying *T. cruzi* based on the distinct amplicon sizes that are recovered from the PCR amplification and subsequent visualization in an agarose gel electrophoresis [7,11,42,52]. However, the highest resolution is obtained by the construction of a phylogenetic tree based on the DNA sequences of the target samples [12]. This phylogenetic analysis involves the additional procedure of sequencing the PCR amplicons. Therefore, using loci with appropriate features for the typification of *T. cruzi* DTUs is an important step in the procedure adopted.

## 3. Traits of a Suitable Genetic Locus for Typifying *T. cruzi*

The molecular markers used in reconstructing phylogenetic trees require them to have particular traits. One of the most important traits is having sufficient synapomorphies (shared and derived DNA polymorphisms), also known as phylogenetic informative characters (PICs) [53]. PICs are DNA polymorphisms shared by at least two individuals within the DNA dataset and are thus considered vital for reconstructing the respective sequences’ evolutionary history and allowing a fully resolved phylogenetic tree to be constructed from the sequences of interest. In other words, they are the number of positions within the DNA sequence alignment that are useful for typifying the genetic lineages of *T. cruzi* (DTUs). The number of PICs needs to be balanced in the genetic locus being considered since there has to be enough DNA sequence conservation between the sequences being compared to ensure homologous sites are appropriately being compared (i.e., a correctly constructed DNA sequence alignment), as well as being conserved enough in the regions where the primers will attach to guarantee the effectivity of the primers across the taxonomic clade intended to be amplified [54,55]. In parallel, the genetic locus should not be too conserved within the amplicon region, or it will not have sufficient PICs to construct a resolved phylogenetic tree.

The phylogenetic tree construction is a powerful taxonomic classification tool; however, it needs to meet some assumptions. In this context, the identification of DNA polymorphisms as derived (apomorphies) or ancestral characters (plesiomorphies) is usually accomplished when an outgroup sequence is included in the analysis, which should preferentially consist of a species that shares relatively recent common ancestry with the ingroup (sequences whose phylogenetic reconstruction is the objective of the study) to qualify as an appropriate outgroup sequence [56,57,58,59]. Thus, in the case of *T. cruzi*, these could be homologous DNA sequences of *T. cruzi marinkellei*, *T. vespertilionis*, *T. rangeli*, or other closely related taxa that have not suffered too much sequence divergence compared to the ingroup. In contrast, *T. brucei*, *Leishmania* spp., or other kinetoplastids with a significant divergence from *T. cruzi* would not be appropriate outgroups.

Furthermore, it would be desirable for the molecular marker of interest to have reference sequences of all *T. cruzi* DTUs available in a public database (e.g., GenBank or TritrypDB), allowing their inclusion in the phylogenetic analysis. Otherwise, a study would have to sequence reference strains to compare the target sequences with the reference DTUs, thus enabling the genetic typification of the target *T. cruzi* sample. Considering that the objective of constructing the phylogenetic tree relies on including reference sequences in the DNA sequence dataset, working with a molecular marker (MM) with several reference sequences a priori is desirable. Therefore, the number and diversity of *T. cruzi* sequences available for a particular MM are advantageous attributes.

An additional key factor to consider in the reconstruction of the evolutionary history of a group of species based on a phylogenetic tree is the use of orthologous sequences (homologous sequences whose divergence dates back to a speciation event) in the analysis [60,61,62]. Failure to do so (i.e., including paralogous sequences) could result in erroneous conclusions since a phylogenetic tree constructed from paralogous sequences (homologous sequences whose divergence dates to a duplication event) will reflect the evolutionary history of the gene and not the species, resulting in the erroneous clustering of sequences [63]. In other words, using MMs that are not part of a gene family with genetic divergence among its gene members is preferable. Fortunately, none of the MMs commonly used to typify *T. cruzi* have relied on gene copies that are suspected to be a part of a gene family. It should be clarified that genetic loci that are found in more than a single copy are not useless; on the contrary, they can be beneficial for diagnostic purposes, as is the case with the minicircle or mini-exon genes, given their high copy number in the kinetoplastid genomes, resulting in their efficacy as molecular markers for the diagnosis of a particular species. Despite this, since DNA polymorphisms can be found within the various copies of the paralogous sequences, their use in phylogenetic inferences is not ideal. In short, the use of orthologous sequences is favored for the reconstruction of the evolutionary history of a particular group of species [61], including the typification of *T. cruzi*.

Finally, a further consideration for an effective molecular marker used to typify a particular species is the size of the molecular marker. Given that the PCR has been the method of choice for amplifying any genetic locus in the typification of *T. cruzi*, it is desirable for the locus to be amplified in a single PCR. In other words, it is desirable for the amplicon size to not be larger than the capacity of the commonly used DNA polymerases [64]. Overall, except for the maxicircle that consists of a concatenation of various mitochondrial genes, all other MMs commonly used to typify *T. cruzi* can be amplified in a single PCR.

In summary, the most suitable loci for typifying *T. cruzi* into DTUs should be analyzed based on having numerous phylogenetically informative characters (synapomorphies), the availability of a sequence that can be used as an outgroup, and an appropriate amount of reference sequences available in public repositories (e.g., GenBank). Therefore, the objective of this review was to provide a quantitative evaluation of the most used genetic locus that has been consistently used for typifying *T. cruzi* into DTUs. Given that the most commonly used loci for the typification of *T. cruzi* are amplified in a single PCR and are not paralogs of diverse gene families, our criteria to evaluate the most appropriate MMs in this review are based on (1) the number of PICs found within each MM; (2) the number of available reference *T. cruzi* sequences in GenBank; and (3) the availability of suitable outgroups.

## 4. Quantitative Approach for the Evaluation of MMs

A bibliography search was performed for published articles that included the keywords “cruzi”, “DTU” and “GenBank” to identify the most common sequenced genetic loci that have been used to typify *T. cruzi* DTUs and whose sequences have been made available through their repository in GenBank. When genetic loci were identified that had been used in multiple studies, the corresponding GenBank accession of one of the reference sequences reported was used as a query for a search of homologous *T. cruzi* sequences (the GenBank accession code used in each dataset is reported in Table 1). The homologous DNA sequence search was conducted with BLAST [65], with default parameters. Based on these search criteria, all homologous sequences that covered a minimum threshold of 90% of the query sequence were downloaded from GenBank. This threshold was set to limit the construction of each dataset to sequences that could be confidently aligned. In the case of the maxicircle dataset, given that this dataset consists of the concatenation of several mitochondrial genes, the query cover threshold was lowered to 85% since most homologous hits were just below the 90% query cover.

All constructed datasets were aligned with MUSCLE in SeaView version 4.0 [66,67]. The minicircle dataset had to be purged of the most divergent sequence regions from the alignment with the use of gBlocks version 0.91b [68] since the sequences were too divergent to construct an alignment confidently. Excluding the minicircle dataset, the homologous *T. cruzi* DNA sequences retrieved made up the DNA alignments of each genetic locus (all constructed alignments were manually checked and included in the Supplementary Material).

A verification of the authenticity of all sequences belonging to *T. cruzi* was performed by constructing a neighbor-joining tree with the inclusion of an outgroup sequence. In most cases, a *T. cruzi marinkellei* homologous sequence was used, with the exception being cases where a *T. c. marinkellei* homologous sequence was not available, meaning that another closely related taxon was used (e.g., *T. conorhini* with Tc24). Within the phylogenetic tree, any sequences that appeared more distantly related (i.e., outside of the monophyletic clade formed by *T. cruzi* sequences) to the ingroup and outgroup were discarded from the dataset since they could have represented misidentified sequences that were erroneously labeled as *T. cruzi* and therefore would have artificially increased the number of PICs estimated from each locus. This verification step could not be carried out for the mini-exon, 5S rRNA, MK or minicircle loci, which have the potential of having slightly higher PICs within their datasets.

To assess the availability of outgroup sequences for each MM, a BLAST search was performed on the NCBI using the representative query DNA sequence (labeled ‘Ac.’ in Table 1), excluding *T. cruzi* sequences. Based on these searches, the closest related species to *T. cruzi* identified was recorded in the column labeled “Out” in Table 1.

The PAUP version 4.0 software package (Swofford, 1991) was used to estimate the number of phylogenetically informative characters (alternatively called parsimony informative sites) within each MM. Table 1 summarizes the measured traits for each genetic locus.

## 5. Highest-Ranking Genetic Loci from the Quantitative Review

According to the bibliographic review performed, 19 genetic loci were identified as the most frequent molecular markers used to typify the genetic background of *T. cruzi*. These genetic loci were quite variable in the range of traits measured: (1) amplicon sizes between 115 and 3681 bp; (2) the number of *T. cruzi* reference sequences publicly available between 32 and 1643; and (3) the number of phylogenetic informative characters constituting 2–176 (Table 1). In total, 4 of the 19 markers are found within the mitochondrial genome, while the remaining loci are found in the nuclear genome, with the top three ranking molecular markers being mitochondrial.

**Figure 1:**
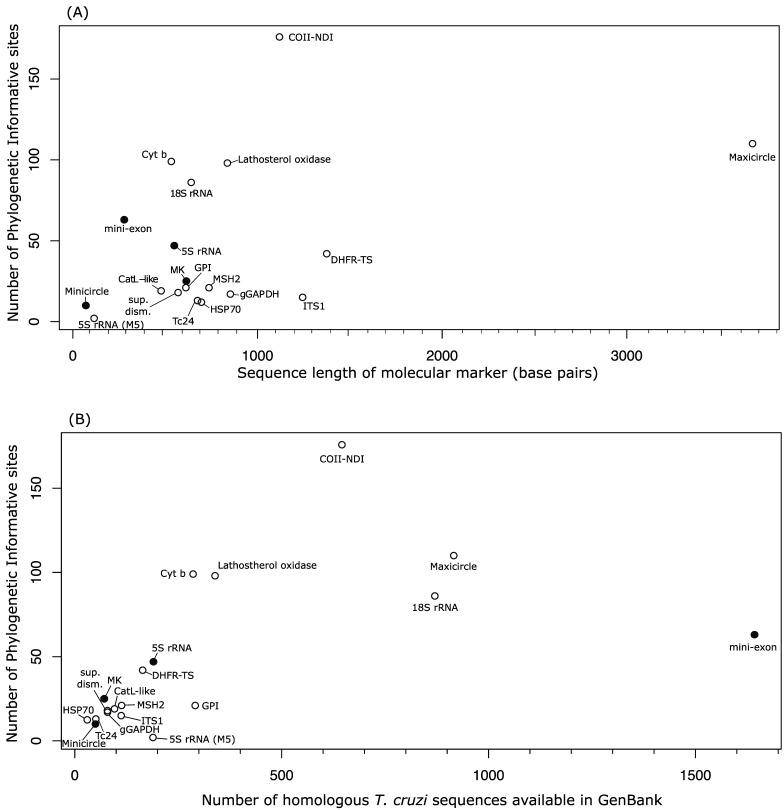
A quantitative evaluation of molecular markers for typifying *Trypanosoma cruzi*. (**A**) The number of phylogenetic informative characters (PICs) identified for the most utilized molecular markers used to typify the genetic background of *Trypanosoma cruzi* lineages. (**B**) The number of *T. cruzi* reference DNA sequences available for each MM as of June 2024. The metrics underscore the variability in phylogenetic informative traits between the most common MMs used to typify *T. cruzi*. Closed circles represent MMs that lack an appropriate outgroup. Open circles represent MMs with appropriate outgroup sequences in public repositories.

**Figure 2:**
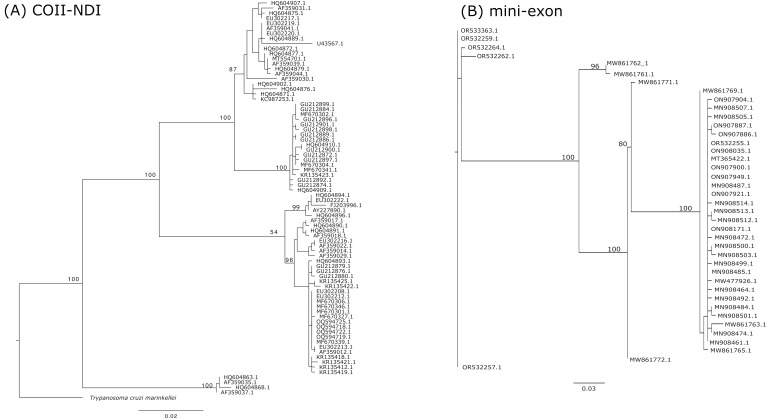
A phylogenetic resolution of the COII-NDI and mini-exon molecular markers to characterize the genetic diversity of *Trypanosoma cruzi.* (**A**) A maximum likelihood tree constructed from the COII-NDI locus. The tree was rooted with *T. cruzi marinkellei*. (**B**) A maximum likelihood tree constructed with the mini-exon locus. Given the absence of an appropriate outgroup for the mini-exon marker, the tree was not rooted. The comparison presents the differences in the phylogenetic resolution obtained from both MMs, underscoring the phylogenetic limitation of an MM with a lower amount of PICs and the lack of having an appropriate outgroup sequence to root the phylogenetic tree. The values above branches represent bootstrap values based on 1000 replicates. Both trees were constructed in W-IQ-TREE (http://www.iqtree.org), using IQ-TREE efficient tree reconstruction [69] and the UFBoot—ultrafast bootstrap approximation [70]. Phylogenetic trees were constructed from a limited number of the available DNA sequences in GenBank. To see the phylogenetic trees that include the totality of DNA sequences available in GenBank, see Supplementary Figure S1.

Based on the quantitatively reviewed criteria, the COII-NDI gene segment is the best locus for *T. cruzi* typing (Table 1 and Figure 1A). This mitochondrial locus codes for the cytochrome c oxidase subunit II and NADH dehydrogenase subunit I proteins, which play a part in the mitochondrial electron transport chain and function in ATP synthesis. Given these enzymes’ vital role, both proteins are ubiquitous in eukaryotes. Nevertheless, a particular trait of kinetoplastids is the ligation of the two genes within the mitochondrial genome. This particular trait is favorable for its use as a molecular marker, given the high abundance of PICs among both genes, resulting in a well-resolved phylogenetic tree (Figure 2A). In *T. cruzi*, the analyzed COII-NDI dataset had a total of 176 PICs. This high number of PICs resulted in COII-NDI having 60% more PICs than the second-ranking locus with the most number of PICs (Table 1), making COII-NDI the top molecular marker in terms of the phylogenetic resolution that can be obtained for the genetic typification of *T. cruzi* based on a single locus. Furthermore, it has a significant amount of available reference *T. cruzi* sequences, ranking number #4 (Figure 1B) as the locus with the most number of reference sequences available in GenBank at the time of this study (645 *T. cruzi* homologous DNA sequences), of which 179 sequences covered the minimum 90% query cover threshold that was set for the construction of each locus dataset (Table 1). Thus, constructing a *T. cruzi* phylogenetic tree with a substantial number of *T. cruzi* reference sequences is achievable with the COII-NDI locus.

Moreover, COII-NDI has a large variety of closely related *Trypanosoma* spp. DNA sequences that can be used as outgroups to root the phylogenetic tree, which is not surprising given the widespread use of the maxicircle in kinetoplastids studies [45,71,72]. Thus, based on the criteria evaluated, the COII-NDI genetic locus is the best option for the genetic typification of *T. cruzi* DTUs.

Following the rank in terms of the number of PICs found within the genetic loci is the maxicircle with 110 PICs (Table 1 and Figure 1A). This genetic locus is unique to kinetoplastids since it is found in the genome that gives kinetoplastids their name (the “kinetoplast”). As a molecular marker, only a fragment of the whole maxicircle has usually been used to typify *T. cruzi* (approximately 3600 bp out of the roughly 20–40 kb that codify the maxicircle DNA sequence). The maxicircle and minicircle are good options for kinetoplastid diagnostic purposes since they have many PICs within the locus typically sequenced [73,74,75]. However, a potential obstacle is the large size of the PCR amplicon, which is obtained by the concatenation of multiple PCRs that target the genes from the maxicircle [8], which might be difficult when funds are limited. However, this molecular marker has a large amplicon size (the largest of all genetic loci typically used to typify *T. cruzi* DTUs), which is part of why it has so many PICs. However, the percentage of PICs along the sequence is quite low, resulting in a percentage of PICs (%PICs; defined as the percentage of the ratio between the number of PICs in an MM and the size of the MM) of only 3% (Table 1). By comparison, most other loci have %PICs much larger than the maxicircle (Table 1). Concerning the number of reference sequences available for this genetic locus and the availability of outgroup sequences, a total of 916 *T. cruzi* homologous sequences were found at the time of the BLAST search (Table 1 and Figure 1B), of which 120 covered the 90% query cover of the concatenated maxicircle sequence that was used as a query (Table 1). This number of references indicates that the maxicircle has an extensive number of available *T. cruzi* reference sequences. As for the accessibility of outgroups, they are mostly available depending on the genetic locus of the maxicircle that is used. But overall, appropriate outgroup sequences are available.

Next on the ranking list are the Cytochrome b (Cyt b) gene segment and the lathosterol oxidase gene with 99 and 98 PICs, respectively (Table 1 and Figure 1A). The Cyt b locus is found within the mitochondrial genome and encodes a protein with the same name that functions within the electron transport chain to produce ATPs. Given its vital function within a cell, the gene can be found in a broad array of eukaryotes and prokaryotes, which has resulted in its popular use as a molecular marker for a wide range of phylogenetic studies within a broad diversity of eukaryotic taxa. The lathosterol oxidase protein is encoded by the SC5D gene (located in the nuclear genome), encoding an enzyme used in the biosynthesis of cholesterol, a metabolic function most commonly found within animals and some protozoans. Both genes practically have the same amount of PICs (99 and 98, respectively) but have slightly different amplicon sizes (approximately 530 vs. 840, respectively), which means Cyt b has a larger %PIC per locus than the Lathostherol oxidase gene (18.5% vs. 11.7%, respectively) (Table 1). Regarding the number of reference sequences available for both loci, Cyt b had 286 homologous *T. cruzi* sequences, of which 252 passed the threshold to be used in our dataset. The Lathostherol oxidase gene had 339 homologous sequences, with 310 covering our threshold (Table 1 and Figure 1B). Furthermore, both molecular markers have DNA sequences of closely related Trypanosomatid species available for their use as outgroups in the corresponding phylogenetic analyses (Table 1), making these loci overall good candidates for their use in the genetic typification of *T. cruzi*.

The gene 18S rRNA trails the previous molecular markers, with 86 PICs from a DNA section of 640 bp in length. The genetic segment that codifies this rRNA is part of the ribosome’s eukaryotic small subunit (40S) and is thus found in all eukaryotes. Due to its ubiquity in eukaryotes and the number of PICs, the 18S rRNA gene has been very commonly used in phylogenetic studies from a wide array of eukaryotic taxa. It therefore has a wide availability of closely related species that can be used as outgroups, as well as an important number of *T. cruzi* sequences in GenBank (Table 1 and Figure 1B). Concerning its utility based on the number of available reference *T. cruzi* sequences, it ranks #3, with a total of 870 homologous *T. cruzi* sequences found in GenBank at the time of the analysis, subtly trailing behind the maxicircle (Figure 1B). However, a potential issue is that upon inspection, the outgroup DNA sequence used for this MM (*T. cruzi marinkellei*) clusters within the ingroup of *T. cruzi* sequences. This potential issue could imply that either the particular outgroup sequences used (ON687560.1 and FJ001664.2) were misidentified and were actually *T. cruzi*, or most likely, some of the sequences currently labeled in GenBank as *T. cruzi* for this MM were the erroneously labeled sequences (i.e., are not *T. cruzi*). This underscores the importance of validating the accuracy of species identification in GenBank sequences, especially when they are used for phylogenetic studies. We recommend performing this validation through a preliminary phylogenetic analysis to ensure sequences cluster within the intended species once a proper outgroup sequence is included.

With respect to this issue, this hypothetical inclusion of non-*T. cruzi* sequences into the 18S rRNA dataset would cause the number of PICs to artificially increase and hence give a falsely overestimated genetic diversity. Nonetheless, the number of *T. cruzi* references available for the 18S rRNA gene makes this genetic locus another suitable candidate for its use in the genetic typification of *T. cruzi*. With 870 *T. cruzi* sequences, 18S rRNA ranks as one the most extensively used molecular markers for *T. cruzi*. Additionally, given the high number of gene copies within the nuclear genome, this genetic locus is easily amplified when working with low-quantity DNA templates.

Subsequently, after the 18S rRNA gene, the SL mini-exon ranks #6 in the list with 63 phylogenetic informative characters (Table 1 and Figure 1A). The biological nature of this MM is quite interesting, given that the mini-exon gene is a trypanosomatid-unique mRNA transcript that is 35 bp long and is spliced to the 5′ end of all mRNAs in most, if not all, trypanosomatid species [7,11,15,76,77,78,79,80]. The functionality of the leader sequence is hypothesized to stabilize the mRNA and provide a recognition site for ribosomes, contributing to ensuring a proper translation for the polycistronic mRNA used by kinetoplastids. As a result of the significant amount of leader sequences that are needed for kinetoplastids to edit all of their mRNAs, the parasite has adapted to have a large number of mini-exon genes composed of tandemly repeated coding blocks [15], within its genome, which in *T. cruzi* is estimated to be approximately 200 copies [81]. This abundance in copies is ideal for any attempts to use it as a diagnostic method for the presence of *T. cruzi*. For instance, targeting a molecular marker that has many copies within the genome of *T. cruzi* is a good option when it is required to typify the lineages of *T. cruzi* from field-collected specimens that do not go through a culture enrichment process of *T. cruzi*. Given that the starting amount of the DNA template used might be low, targeting a high-copy genetic locus increases the likelihood of effectively amplifying the PCR-targeted sequence. Ricardo P. Souto and colleagues proposed using this unique DNA sequence of kinetoplastids to typify *T. cruzi* strains in 1996 [11]. Since then, it has been widely used, and it is therefore not surprising for it to have become the most commonly used MM for the typification of *T. cruzi*, which is why it is the MM with the largest number of *T. cruzi* sequences currently available in GenBank, with a total of 1643 at the time of this study (Table 1 and Figure 1B).

Additionally, as mentioned previously, the number of PICs found within the mini-exon is not insignificant (63 sites) (Table 1), and the small size of this PCR amplicon explains how this MM has the largest percentage of PICs per locus (%PICs) of all the genetic loci analyzed (22.6%) (Table 1). Nonetheless, one of its major disadvantages is the inability to include proper outgroup sequences for this marker in the corresponding phylogenetic analyses. The reason appears to be the high divergence within the DNA mini-exon sequence among kinetoplastid species and within *T. cruzi* lineages, which is why it was proposed as a molecular marker in the first place. Since it appears to have significant differences in the amplicon length between lineages, it has been used to typify *T. cruzi* strains based solely on the size amplified via the PCR [11,82]. Henceforth, this extreme polymorphism hinders the ability to properly identify the homologous sequences in closely related taxa, resulting in the failure to include proper outgroups for the rooting of a phylogenetic analysis and thus interfering with the ability to reconstruct a well-resolved phylogenetic tree (Figure 2B). These excessive polymorphisms are evidenced by the fact that exhaustive homologous sequence searches with BLAST fail to identify homologous sequences that cover the entirety of the query and only find homologous sequences that cover <25% of the query coverage. The limitation of including outgroup DNA sequences is not trivial since using outgroup sequences is vital for adequately rooting a phylogenetic tree. Even though these molecular markers are useful to typify the major genetic lineages of *T. cruzi* by analyzing the amplicon sizes, their use for phylogenetic analyses (i.e., a higher resolution to determine the diversity within a DTU) is not recommended.

Another potential outcome of the inability to include an outgroup with the mini-exon dataset was an overestimated number of PICs for this MM due to the failure to complete the second checkpoint to guarantee the exclusion of not-*T. cruzi* DNA sequences. This second checkpoint was not accomplished because it required the use of an outgroup.

In positions #7 and #8, trailing behind the mini-exon, are the 5S rRNA and dihydrofolate reductase–thymidylate synthase (DHFR-TS) loci, with 47 and 42 phylogenetic informative characters, respectively. While 5S rRNA codifies a ribosomal RNA that forms part of the small ribosomal subunit (which means this gene is pretty much universal to all eukaryotes), the DHFR-TS gene codifies a bifunctional protein that plays a vital role in the replication of DNA and is unique to Trypanosomatids and other protozoa and has thus been proposed as a potential drug target. This bifunctional enzyme plays a role in reducing dihydrofolate to tetrahydrofolate within the folate cycle, which is essential for synthesizing nucleotides. Meanwhile, the thymidylate synthase enzyme function is needed to catalyze the conversion of dUMP to dTMP within the cycle of thymine synthesis, and thus, it is vital for DNA replication. The first phylogenetic tree published with the 5S rRNA locus in the reconstruction of the evolutionary history of *T. cruzi* was conducted by Westenberger et al. [83], while Machado and Ayala reported the first phylogenetic analysis for the DHFR-TS locus [45]. Both genetic loci have practically the same number of reference sequences available in GenBank (190 and 164, respectively) (Figure 1B), although the respective datasets were significantly different between each molecular marker, given the scarcity of homolog *T. cruzi* sequences that covered the minimum 90% query cover threshold that was set for the construction of each locus (Table 1). Conversely, appropriate outgroup sequences were only available for the DHFR-TS. The 5S rRNA locus does not have suitable outgroup sequences that cover more than 30% of the *T. cruzi* %S rRNA sequences and thus has this critical limitation as an MM.

Following the DHFR-TS gene are the remaining eleven loci, forming a cluster of genes with a significantly lower number of PICs (Figure 1A) than the rest. Within this cluster are the following genes: mevalonate kinase (MK), MutS Homolog 2 (MSH2), Glucose-6-phosphate isomerase (GPI), cathepsin L-like (CatL-like), superoxide dismutase, glycosomal glyceraldehyde phosphate dehydrogenase (gGAPDH), internal transcribed spacer 1 (ITS1), the trypomastigote excretory–secretory protein (Tc24), heat-shock protein 70 (HSP70), minicircle and the 5S rRNA (M5) loci. With PICs ranging between 2 and 25, respectively (Table 1), the number of reference sequences available in GenBank for each locus was also significantly smaller than for the previously discussed loci analyzed (Figure 1B), perhaps as a result of the low resolution that their use can accomplish. Although the majority of these loci have outgroup sequences available in GenBank, the low amount of reference sequences and, most importantly, the low number of phylogenetically informative characters justify the exclusion of their use as individual molecular markers used to typify the genetic diversity of *T. cruzi*.

A particular reference to the minicircle is needed since the extreme sequence divergence within this marker does not allow reliable DNA sequence alignments. Consequently, divergent DNA alignment sections were removed with gBlocks, resulting in a smaller dataset. While this reduces the number of PICs, retaining unreliable DNA alignments would unmistakably result in an erroneous phylogeny [68]. Additionally, the lack of an outgroup sequence (Table 1) further supports the exclusion of the minicircle as a suitable molecular marker for typifying *T. cruzi* based on a phylogenetic reconstruction.

Therefore, as a whole, the phylogenetic robustness that can be obtained from molecular markers that fulfill all three major traits evaluated in this review (a high number of phylogenetic informative characters, a significant amount of *T. cruzi* reference sequences and the availability of appropriate outgroup sequences) can be clearly observed in Figure 2. This figure juxtaposes the maximum likelihood phylogenetic trees of the best-ranked genetic locus versus one that does not have an outgroup (COII-NDI and the mini-exon, respectively). The divergence in the branch lengths and clustering positions of sequences identified with the COII-NDI phylogeny, as well as the importance of rooting the tree with an appropriate outgroup, underscores the need to carefully consider the parameters used in a quantitative review for the proper selection of MMs to typify *T. cruzi*.

## 6. Concluding Remarks

Although sequencing technologies are becoming more accessible, PCR amplicons remain essential for the genetic typing of *T. cruzi*, especially in understanding crucial aspects of its biology. Selecting an appropriate molecular marker (MM) depends heavily on the research question. For instance, detecting intra-DTU differences often requires loci with sufficient DNA polymorphisms that will allow the reconstruction of a detailed phylogenetic tree. To address these considerations, we provide a quantitative review of the most frequently employed genetic loci, evaluating them on three key criteria: (1) the number of phylogenetic informative characters; (2) the availability of *T. cruzi* reference sequences in public repositories; and (3) the accessibility of DNA sequences from related taxa for an outgroup comparison. This effort identified nineteen genetic loci as the most commonly applied for the genetic typification of *T. cruzi* strains. Of the nineteen loci analyzed, COII-NDI ranked highest due to its extensive outgroup sequence availability, abundant reference sequences for *T. cruzi*, and high number of phylogenetically informative characters—60% more than the next marker. The utility of fulfilling these traits can be clearly observed in Figure 2, where the phylogenetic resolution obtained from COII-NDI clearly is much higher than the mini-exon tree, which has a significantly lower number of PICs and does not have an appropriate outgroup sequence.

As a whole, mitochondrial markers, including the maxicircle and the Cytochrome b loci, are also highly effective markers to typify *T. cruzi*. Among the nuclear loci, lathosterol oxidase and 18S rRNA ranked best. When feasible, we recommend using a combination of these top-ranking loci for comprehensive genetic typing. If limited to a single locus, COII-NDI is the most suitable option.

## Figures and Tables

**Table 1:** Summary of traits for the molecular markers most utilized for typifying *Trypanosoma cruzi*.

MM	bp	n	nG	PICs	% PICs	Acc. No.	OS	GL
COII-NDI	1119	179	645	176	15.72	HQ604893.1	T. c. m.	MT
Maxicircle	3681	120	916	110	2.98	KP136828.1	sev.	MT
Cyt b	533	252	286	99	18.57	HQ713679.1	T. c. m.	MT
Lathosterol oxidase	837	310	339	98	11.70	JN050587.1	T. c. m.	NUC
18S rRNA	641	348	870	86	13.41	LT220268.1	T. c. m. *	NUC
mini-exon	278	1138	1643	63	22.66	AM259467.1	NA	NUC
5S rRNA	550	16	190	47	8.54	M59503.1	NA *	NUC
DHFR-TS	1375	98	164	42	3.05	MF784874.1	T. c. m.	NUC
MK	615	47	71	25	4.06	MH671655.1	NA	NUC
MSH2	737	116	113	21	2.84	MF074182.1	T. r.	NUC
GPI	612	61	291	21	3.43	KT737478.1	T. c. m.	NUC
CatL-like *	478	71	96	19	3.97	JF421288.1	T. c. m.	NUC
Superoxide dismutase (sup. dism.)	570	11	79	18	3.15	XM_808937.1	T. r.	NUC
gGAPDH	853	23	79	17	1.99	OQ095397.1	T. c. m.	NUC
ITS1	1244	6	112	15	1.20	AF362831.1	T. r.	NUC
Tc24	675	49	51	13	1.92	OL781152.1	T. con.	NUC
HSP70	696	27	32	12	1.72	ON653039.1	T. c. m.	NUC
Minicircle *	71	48	50	10	14.08	AJ747914.1	Vert.	MT
5S rRNA (M5)	115	168	189	2	1.73	XR_008255252.1	T. r.	NUC

MM: molecular marker. bp: number of nucleotides included in DNA sequence alignment dataset. n: number of *T. cruzi* sequences included in dataset. nG: number of homologous sequences available of *T. cruzi* in GenBank as of 6 September 2024. PICs: number of SNPs that are phylogenetically informative characters. %PICs: percentage of dataset with phylogenetic informative characters. Acc. No.: GenBank accession code of sequence used as a query to create dataset. OS: closest related outgroup species. GL: genomic location. MT: located in mitochondrial genome. NUC: located in nuclear genome. T. c. m.: *Trypanosoma cruzi marinkellei*. T. r: *Trypanosoma rangeli*. T. con: *Trypanosoma conorhini*. sev: several different closely related species, but varies given the gene section analyzed since the maxicircle MM is concatenation of different loci. Vert: only found vertebrate DNA. NA: not available. NA *: not available that cover >90% of query. Minicircle *: dataset had the divergent regions removed with gBlocks (see methods for justification). T. c. m. *: The outgroup sequence clusters within the *T. cruzi* ingroup sequences, potentially reflecting that either some *T. cruzi* labeled sequences in GenBank are misidentified, or the outgroup sequence is misidentified. This could be overestimating the number of PICs. CatL-like *: several sequences labeled as *T. cruzi* were removed from the dataset since they appear to be misidentified (sequences removed: MZ087227.1, MZ087228.1, M90067.1, MZ087241.1, MZ087239.1, MZ087242.1, MZ087240.1, MZ087238.1, MZ087237.1, XM_797951.1, MZ087236.1, JF421346.1).

## Data Availability

No new data were created or analyzed in this study. The data used for this review was publicly available in GenBank, it was actively analyzed as part of our study.

## Supplementary Materials

The following supporting information can be downloaded at: https://www.mdpi.com/article/10.3390/pathogens14010072/s1, Figure S1: Comparison of phylogenetic trees (COII-NDI & mini-exon) constructed with all *T. cruzi* available sequences in GenBank as of July 2024.
