# Supplementary material for: Molecular Markers for the Phylogenetic Reconstruction of Trypanosoma cruzi: A Quantitative Review
Source: Pathogens. 2025 Jan 14;14(1):72. doi: 10.3390/pathogens14010072 (PMC11768369; doi:10.3390/pathogens14010072)
Supplement: Supplementary file 1 [file pathogens-14-00072-s001.zip › Supplementary_Figure_S1_Legend.pdf]

Supplementary Figure S1. Comparison of phylogenetic trees (COII-NDI & mini-exon) constructed with all *T. cruzi* available sequences in GenBank as of July 2024.
(A) Maximum likelihood tree constructed from the COII-NDI locus rooted with *T. cruzi marinkellei*. (B) Maximum likelihood tree constructed with the mini-exon locus. The absence of an appropriate outgroup for the mini-exon marker, hinders the possibility of rooting the tree. The values above branches represent bootstrap values based on 1,000 replicates. Both trees were constructed in IQ-TREE (http://www.iqtree.org), using IQ-TREE Efficient Tree Reconstruction [69] and the UFBoot-Ultrafast Bootstrap Approximation [70].
