# Supplementary figures and images for: Molecular Markers for the Phylogenetic Reconstruction of Trypanosoma cruzi: A Quantitative Review
Source: Pathogens. 2025 Jan 14;14(1):72. doi: 10.3390/pathogens14010072 (PMC11768369; doi:10.3390/pathogens14010072)

(A) COII-NDI

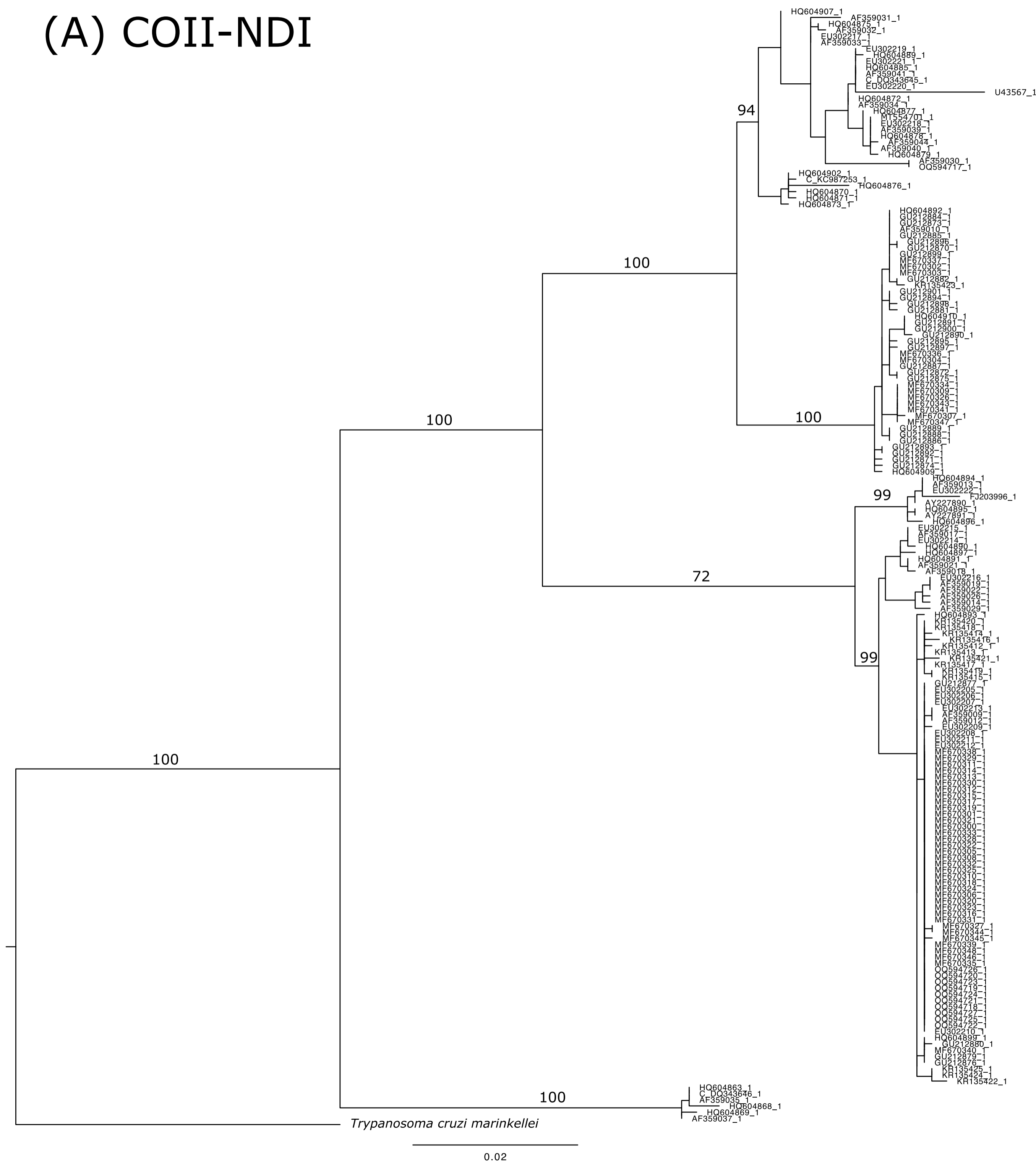

(B) mini-exon

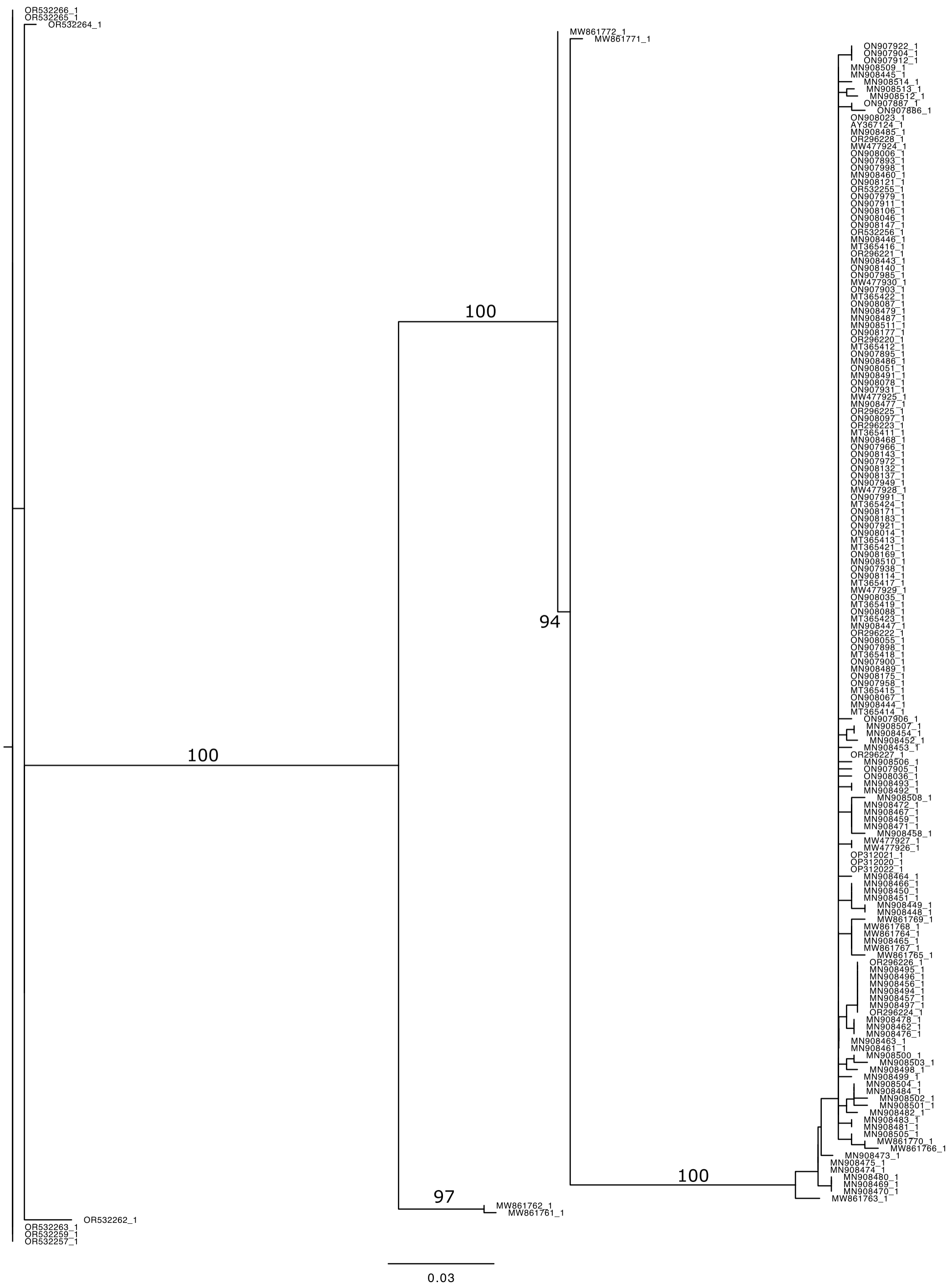

Supplement: Supplementary file 1 [file pathogens-14-00072-s001.zip › Supplementary_Figure_S1.pdf]
